# Surfactant protein D (*SP*-*D*) gene polymorphism rs721917 is an independent predictor of acute kidney injury development in sepsis patients: a prospective cohort study

**DOI:** 10.1186/s13613-019-0617-5

**Published:** 2020-01-13

**Authors:** Jiao Liu, Jianying Yao, Lidi Zhang, Yizhu Chen, Hangxiang Du, Zhenliang Wen, Dechang Chen

**Affiliations:** 1 Department of Critical Care Medicine, Shanghai Jiaotong University, School of Medicine, Ruijin Hospital North, Shanghai, 201801 China; 2 Intensive Care Unit, The First People’s Hospital of Kunshan, Kunshan, 215300 China

## Abstract

**Background:**

Currently, there are no reliable predictors of risk of development and severity of acute kidney injury (AKI) in septic patients. The surfactant protein D (SP-D) polymorphism rs721917C/T is associated with a greater susceptibility to AKI in the Chinese population. Our aim was to evaluate the value of SP-D polymorphisms rs721917C/T and of plasma SP-D levels to predict the risk of development of AKI (defined with KDIGO criterion) in septic patients.

**Methods:**

The study enrolled septic patients admitted to the Critical Care Department of two tertiary care hospitals. *SP*-*D* rs721917C/T polymorphisms were determined using the PCR-SSP method. Plasma SP-D and urine NGAL contents were measured using commercially available ELISA kits.

**Results:**

330 septic patients were included. Their SOFA scores were 12 ± 3. Patients with AKI (*n* = 156) had higher plasma SP-D levels (median: 153 ng/mL, range 111–198 ng/mL) and urinary NGAL levels (median: 575 ng/mL, range 423–727 ng/mL) than those without AKI (SP-D median: 124 ng/mL, range 81–159 ng/mL, *P *= 0.001; NGAL median: 484 ng/mL, range 429–573 ng/mL). Plasma SP-D levels of AKI patients were correlated with urinary NGAL contents (*r *= 0.853). In 32 patients receiving continuous renal replacement therapy (CRRT), plasma SP-D levels correlated with duration of CRRT (*r *= 0.448). The area under the receiver operating characteristic curve for plasma SP-D levels to predict AKI was 0.84. Patients with AKI had a higher rate of rs721917 CC genotype (AKI: 35% vs. non-AKI: 20%; *P *= 0.012), but a significantly lower rate of TT genotype (AKI: 19% vs. non-AKI: 26%; P = 0.005). SP-D rs721917 CC genotype was an independent predictor of AKI (*P* = 0.044) and mortality (*P* = 0.014).

**Conclusion:**

Our study showed that increased plasma SP-D level is associated with a higher risk of AKI in patients with sepsis. The SP-D rs721917CC genotype is an independent and significant predictor of AKI development and mortality of septic patients. The SP-D rs721917C/T polymorphisms should be further studied as diagnostic and prognostic biomarkers to facilitate early recognition of AKI.

## Background

Acute kidney injury (AKI) is a serious and frequent complication of sepsis and is present in approximately 51–64% of patients with sepsis, which remains the most common cause of AKI in critically ill patients [1]. The presence of AKI has contributed a sharp increase in mortality in intensive care unit (ICU) patients, particularly those with sepsis [2]. This high mortality associated with AKI in septic patients may be partially explained by delayed appreciation and diagnosis of AKI, which currently is based on elevated serum creatinine levels or the presence of oliguria. These two markers have limited sensitivity and specificity for early recognition of renal dysfunction, necessitating the development of new biomarkers that allow earlier diagnosis and risk stratification of AKI in septic patients.

Today, there are no reliable predictors of risk of development and severity of AKI in septic patients. Surfactant protein D (SP-D) is an innate immune defense molecule of the collectin family, and is expressed in the lungs and extrapulmonary epithelia including tissues in the urinary tract. SP-D has immune modulatory effects depending on its oligomerization [3]. In kidney diseases, SP-D plays a key role in activating the humoral arm of innate immunity and host defense [4]. The biological functions and clinical relevance of SP-D have just started to be elucidated. Xie et al. found that circulating SP-D levels were associated with the 1-year prognosis of chronic kidney disease patients [5]. On the other hand, Liu et al. found that SP-D was not associated with an increased risk of mortality of acute lung injury patients [6]. ICU patients with AKI have a twofold higher mortality versus ICU patients without AKI [7]. A structural single nucleotide polymorphism rs721917 in the *SP*-*D* gene, known as Met11Thr, alters the plasma levels of SP-D. Horimasu et al. found that SP-D polymorphisms rs721917C/T and rs2243639A/G were significantly and independently correlated with plasma SP-D levels in Germans with interstitial lung diseases [8]. Our previous study also revealed that higher plasma SP-D levels were associated with a more adverse clinical outcome and Chinese patients carrying SP-D rs721917C/T had greater susceptibility to AKI [9].

Since it is difficult to predict subsequent development of AKI in septic patients, we conducted a prospective cohort study to determine plasma SP-D levels and *SP*-*D* polymorphisms rs721917C/T and rs2243639A/G of septic patients with AKI versus those without AKI. We wanted to analyze if plasma SP-D levels and *SP*-*D* polymorphisms are associated with subsequent development and severity of AKI as well as outcome using clinical data of septic patients in the Department of Critical Care Medicine (CCMD) at two tertiary care centers in China.

## Patients and methods

### Patients

This prospective study was carried out at CCMD of Renmin Hospital of Wuhan University, Wuhan, China, and the Intensive Care Unit of The First People’s Hospital, Kunshan, Jiangsu, China, between March, 2015 and June 2017. The study protocol was approved by the local ethics committee of the authors’ affiliated hospitals and patients provided written informed consent to the study.

We included adult septic or septic shock patients aged between at least 18 years and 70 years. We excluded pregnant or lactating women and patients with chronic kidney disease and chronic respiratory disease. Patients with missing age, gender, admission and discharge diagnoses were also excluded. The third international consensus definitions for sepsis and septic shock were used [10]. Acute kidney injury (AKI) was defined and classified according to the Kidney Disease Improving Global Outcomes (KDIGO) guidelines into stages 1–3 [11]. AKI was defined as serum creatinine (Scr) elevation exceeding 0.3 mg/dL (26.5 mol/L) within 48 h, or serum creatinine elevation exceeding 1.5-fold of the baseline value, or urine output less than 0.5 mL/kg/h for more than 6 h. Acute respiratory distress syndrome (ARDS) was diagnosed according to the 2012 Berlin criteria [12]. Baseline creatinine was defined as creatine level on admitting.

### Patient evaluation

We collected patient demographic and baseline data, pre-existing morbidities including hypertension and diabetes, SOFA scores, daily net fluid balance, PaO_2_/FiO_2_ ratio, extracorporeal membrane oxygenation therapy, pulmonary function variables (mean airway pressure, positive end-expiratory pressure, compliance of the respiratory system), necessity of continuous renal replacement therapy (CRRT), use and dosages of vasoactive drugs, blood chemistry values, and blood microbiological culture results. Concurrence with ARDS or AKI was also recorded. Patients were categorized daily for KDIGO stages on the basis of serum creatinine concentration and urine output. The worst of both values was applied for AKI staging. Sources of infection including the respiratory tract, the urinary tract, bloodstream, the abdominal cavity or unknown sites were documented. The observation period was defined from admission into the CCMD to leaving the CCMD or the date of death.

### DNA genotyping

Five milliliters of peripheral venous blood was obtained from septic patients. After centrifugation at 5000 rpm/min for 5 min, leucocytes were collected, aliquoted and stored at − 80 °C until processing. DNA was then extracted from the isolated peripheral leukocytes using commercially available kits (Solarbio, Beijing, China) as previously described [13]. *SP*-*D* rs721917C/T and rs2243639A/G polymorphisms were determined using the PCR-SSP method. PCR was run at 94 °C for 1 min followed by 5 cycles of 94 °C for 20 s, 65 °C for 65 s, and 72 °C for 25 s and then was run at 94 °C for 25 s, 55 °C for 50 s, and 72 °C for 30 s for totally 21 cycles followed by 4 cycles of 94 °C for 30 s, 50 °C for 60 s, and 72 °C for 120 s with a final extension at 72 °C for 3 min. The PCR products were resolved by 2% agarose gel electrophoresis.

### ELISA

Five milliliters of peripheral venous blood and 10 mL urine were obtained from septic patients. After centrifugation at 5000 rpm/min for 5 min, plasma was collected and stored at − 80 °C until processing. Plasma SP-D and urine NGAL contents were examined using commercially available ELISA kits (R&D Inc, Minneapolis, MN, USA) as previously described [14].

### Statistical analysis

Numerical data were expressed in mean ± SD or median (interquartile ranges, IQR), and categorical data were expressed in number and percentage. Genetic equilibrium of allele and genotype frequency was evaluated using Hardy–Weinberg equilibrium (HWE); if *P *> 0.05, the frequency of allele and genotype was considered in genetic equilibrium. Comparison of categorical data was done using Pearson’s two-tailed Chi-squared test or Fisher exact test (for sample number < 5 in a group). Continuous variables among multiple groups (age, SOFA scores, plasma SP-D and urinary NGAL levels), if they passed variance homogeneity test, were compared using one-way ANOVA test; if they failed variance homogeneity test, Kruskal–Wallis test was used. Comparison of continuous variables between two groups was done using the SNK q test. Multivariate logistic regression analysis and rank order logistic regression analysis were performed to study correlation between variables and AKI development, AKI stage and mortality, and odd ratios (ORs) were calculated. Pearson correlation analysis was undertaken to study whether plasma SP-D levels and urinary NGAL levels correlated with AKI stage and duration for CRRT. The area under the receiver operating characteristic (ROC) curves for the ability of SP-D and NGAL to distinguish septic AKI patients from the non-AKI ones were calculated.

All analyses were conducted at a two-sided alpha error level of 5%. All analyses were performed using SPSS (version 21, IBM, Chicago, IL, USA). For graphical presentations GraphPad Prism 7 (Graph-Pad, San Diego, CA, USA) was used.

## Results

### Patient demographic and baseline characteristics

**Fig. 1:**
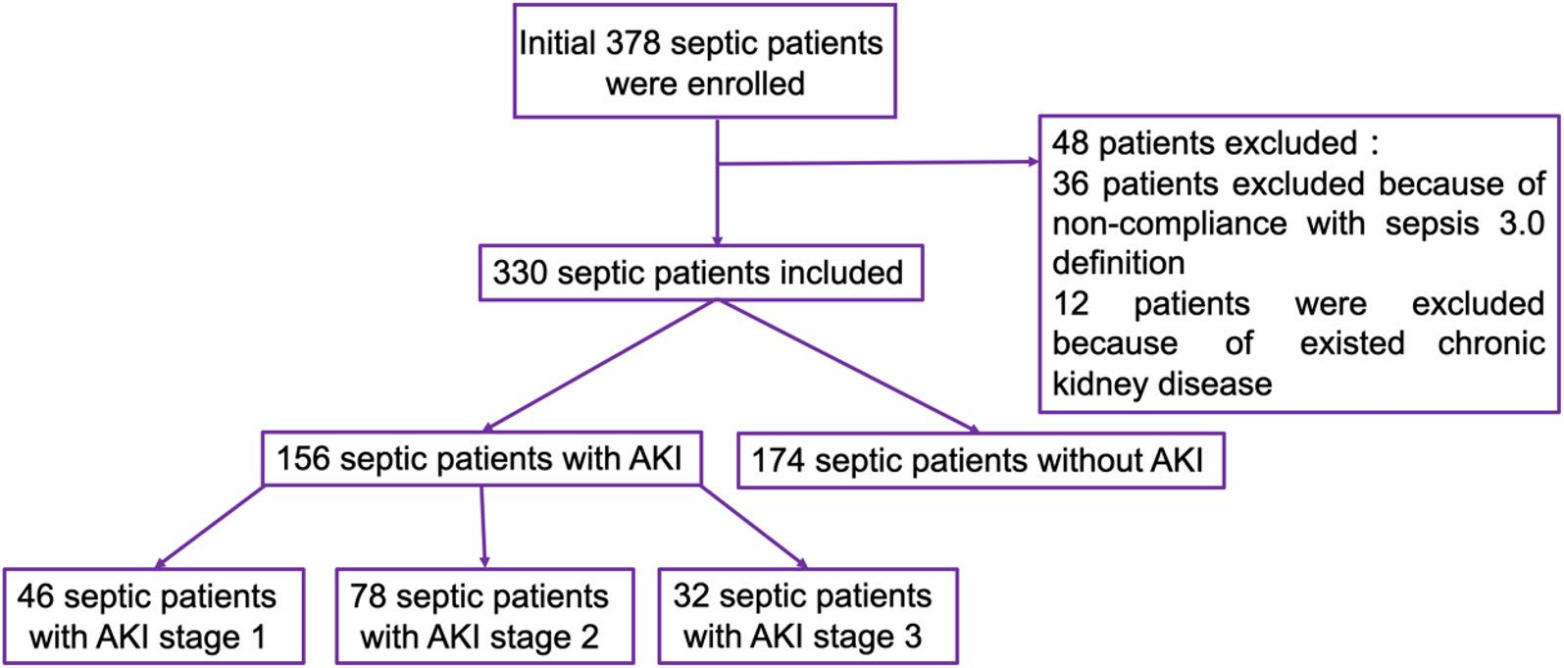
The study flowchart

The study flowchart is shown in Fig. 1. During the study period, 378 patients were diagnosed with sepsis and admitted to the CCMD of the two hospitals. Thirty-six patients who did not meet the third international consensus definitions for sepsis and septic shock were excluded; 12 patients with preexisting chronic kidney disease were also excluded. Finally, 330 septic patients including 121 patients with septic shock were eligible for the study. Patient demographic and baseline characteristics are shown in Table 1. The study population included 158 males and 172 females. Their mean age was 47 ± 8 years (range 20–68 years). One hundred fifty-six (47%) patients developed AKI during their stay in the CCMD: patients with 46 KDIGO stage 1, 78 patients with KDIGO stage 2, and 32 patients with KDIGO stage 3. Seventy-five (23%) patients had ARDS. The mean SOFA scores of the study population was 12 ± 3 (range 8–21). The median plasma SP-D content for the whole study population was 137 ng/mL (range 81–199 ng/mL) (reference plasma SP-D values for normal subjects: 57–174 ng/mL).

**Table 1:** Demographic and baseline characteristics of the study population

Variables	Sepsis			*P*
	All	Non-AKI	AKI	
N	330	174	156	
Mean age, years (SD)	47 ± 8	44 ± 7	50 ± 7	0.013^a^
Male sex, *n*(%)	158 (48)	83 (48)	75 (48)	0.750
Mean SOFA scores (SD)	12 ± 3	10 ± 4	12 ± 3	0.060
Positive blood culture, *n*(%)	69 (21)	29 (17)	40 (26)	0.145
Shock, *n*(%)	121 (37)	48 (28)	73 (47)	0.002^a^
ARDS, *n*(%)	102 (31)	60 (35)	42 (27)	0.089
Comorbidities, *n*(%)				
Hypertension	45 (14)	20 (12)	25 (16)	0.231
Diabetes mellitus	53 (16)	22 (13)	31 (20)	0.074
Infection foci, *n* (%)				
Respiratory system	142 (43)	75 (43)	67 (43)	0.731
Abdominal cavity	55 (17)	31 (18)	24 (15)	0.554
Urinary tract	71 (22)	47 (27)	24 (15)	0.010^a^
Bloodstream	55 (17)	17 (10)	38 (24)	0.010^a^
Unknown	7 (2)	4 (2)	3 (2)	0.813
Average plasma SP-D, ng/mL	137 ± 25	124 ± 20	153 ± 22	0.001^a^
Median (IQR) urinary NGAL, ng/mL	501 (477, 560)	480 (469, 496)	561 (536, 611)	0.001^a^

^a^Compared with no AKI patients, *P *< 0.01

### Characteristics of septic patients with AKI versus those without AKI

Patient demographic and baseline characteristics stratified by AKI are shown in Table 1. The mean age of patients with AKI (50 ± 7 years) was significantly lower than those without AKI (44 ± 7 years; *P *= 0.013). Septic shock was more frequent in patients with AKI (47%) than in those without AKI (28%; *P *= 0.002). In addition, significantly more (24%) septic patients with AKI had bloodstream infections than those without AKI (10%; *P *= 0.01). Patients with AKI had significantly higher plasma SP-D levels (median: 150 ng/mL, range 138–160 ng/mL) than those without AKI (median: 101 ng/mL, range 82–111 ng/mL; *P *= 0.01).

### SP-D polymorphisms rs721917C/T and rs2243639A/G of septic patients with AKI vs. those without AKI

SP-D polymorphisms rs721917C/T and rs2243639A/G of septic patients with AKI vs. those without AKI are shown in Table 2. Patients with AKI had a significantly higher rate of rs721917 CC genotype (AKI: 35% vs. non-AKI: 20%; *P *= 0.012), but a significantly lower rate of TT genotype (AKI: 19% vs. non-AKI: 26%; *P* = 0.005). In addition, patients with AKI had a significantly higher rate of rs721917C allele than those without AKI (58% vs. 47%, respectively; *P *= 0.05). There was no significant difference between AKI patients vs. non-AKI patients in terms of rate of rs2243639 CC, CT and TT genotypes and in terms of rate of rs2243639 A and G allele.

**Table 2:** *SP*-*D* polymorphisms in the study population

SP-D polymorphisms	All	Sepsis		*p*	Sepsis		*p*
		Non-AKI	AKI		Non-survivors	Survivors	
N	330	174	156		78	252	
rs721917 genotypes							
CC	89 (27)	35 (20)	54 (35)	0.003^$$^	42 (54)	47 (19)	0.000**
CT	167 (51)	94 (54)	73 (47)	0.190	28 (36)	139 (55)	0.003**
TT	74 (22)	45 (26)	29(19)	0.114	8 (10)	66 (26)	0.030*
rs721917 alleles							
C	345 (52)	164 (47)	181 (58)	0.048^$^	112 (72)	233 (46)	0.001**
T	315 (48)	184 (53)	131 (42)		52 (33)	277 (55)	
rs2243639 genotypes							
AA	52 (16)	27 (16)	25 (16)	0.899	12 (15)	38 (15)	0.948
AG	142 (43)	81 (46)	61 (39)	0.172	36 (46)	106 (42)	0.524
GG	136 (41)	66 (38)	70 (45)	0.201	30 (39)	108 (43)	0.492
rs2243639 alleles							
A	246 (37)	135 (39)	111 (36)	0.546	60 (39)	182 (36)	0.707
G	414 (63)	213 (61)	201 (64)		96 (62)	322 (64)	

** Compared with non-survivors, *P *< 0.01

^$^Compared with no AKI patients, *P *< 0.05

^$$^Compared with no AKI patients, *P *< 0.01

In addition, SP-D polymorphisms rs721917 genotypes (CC, CT and TT) and alleles (C, T), and rs2243639 genotypes (AA, AG and GG) and alleles (A, G) were validated by HWE (*P *> 0.05 in all), indicating genotype and allele distribution was in genetic equilibrium.

### SP-D polymorphisms rs721917C/T and rs2243639A/G and AKI KDIGO stages

SP-D polymorphisms rs721917C/T and rs2243639A/G in septic patients with KDIGO stage 1, 2 and 3 are shown in Table 3. The frequency of SP-D rs721917 CC genotype and C allele in KDIGO stage 3 patients was significantly higher than that of KDIGO stage 1 patients (*P *< 0.05 in both), meanwhile, the frequency of rs721917TT genotype and T allele in KDIGO stage 3 patients was significantly lower than that of KDIGO stage 1 patients (*P *< 0.05 in both). However, there was no statistical difference in the frequency of SP-D rs721917CC genotype and C allele between KDIGO stage 1 and 2 patients (*P *> 0.05 in both), and between KDIGO stage 2 and 3 patients (*P *> 0.05 in both). Furthermore, there was no statistical difference in rs2243639 AA, AG and GG genotype and the frequency of A, G allele among KDIGO stage 1, 2, and 3 patients (*P *> 0.05).

**Table 3:** *SP*-*D* polymorphisms in sepsis patients according to AKI KDIGO stage

SP-D polymorphisms	AKI KDIGO stages			*P*
	1	2	3	
*N*	*N* = 46	*N* = 78	*N* = 32	
rs721917 genotypes				
CC	7 (15)	31 (40)	16 (50)	0.003**
CT	29 (63)	32 (41)	12 (38)	0.030*
TT	10 (22)	15 (19)	4 (12)	0.577
rs721917 alleles				
C	43 (47)	94 (60)	44 (69)	0.017*
T	49 (53)	62 (40)	20 (31)	
rs2243639 genotypes				
AA	7 (15)	12 (15)	6 (19)	0.895
AG	16 (35)	33 (42)	12 (38)	0.694
GG	23 (50)	33 (42)	14 (42)	0.700
rs2243639 alleles				
A	30 (33)	57 (37)	24 (38)	0.771
G	62 (67)	99 (63)	40 (62)	

* Compared with TT genotype, *P *< 0.05

** Compared with TT genotype, *P *< 0.01

### Plasma SP-D levels, urinary NGAL levels and prognosis of septic patients

**Fig. 2:**
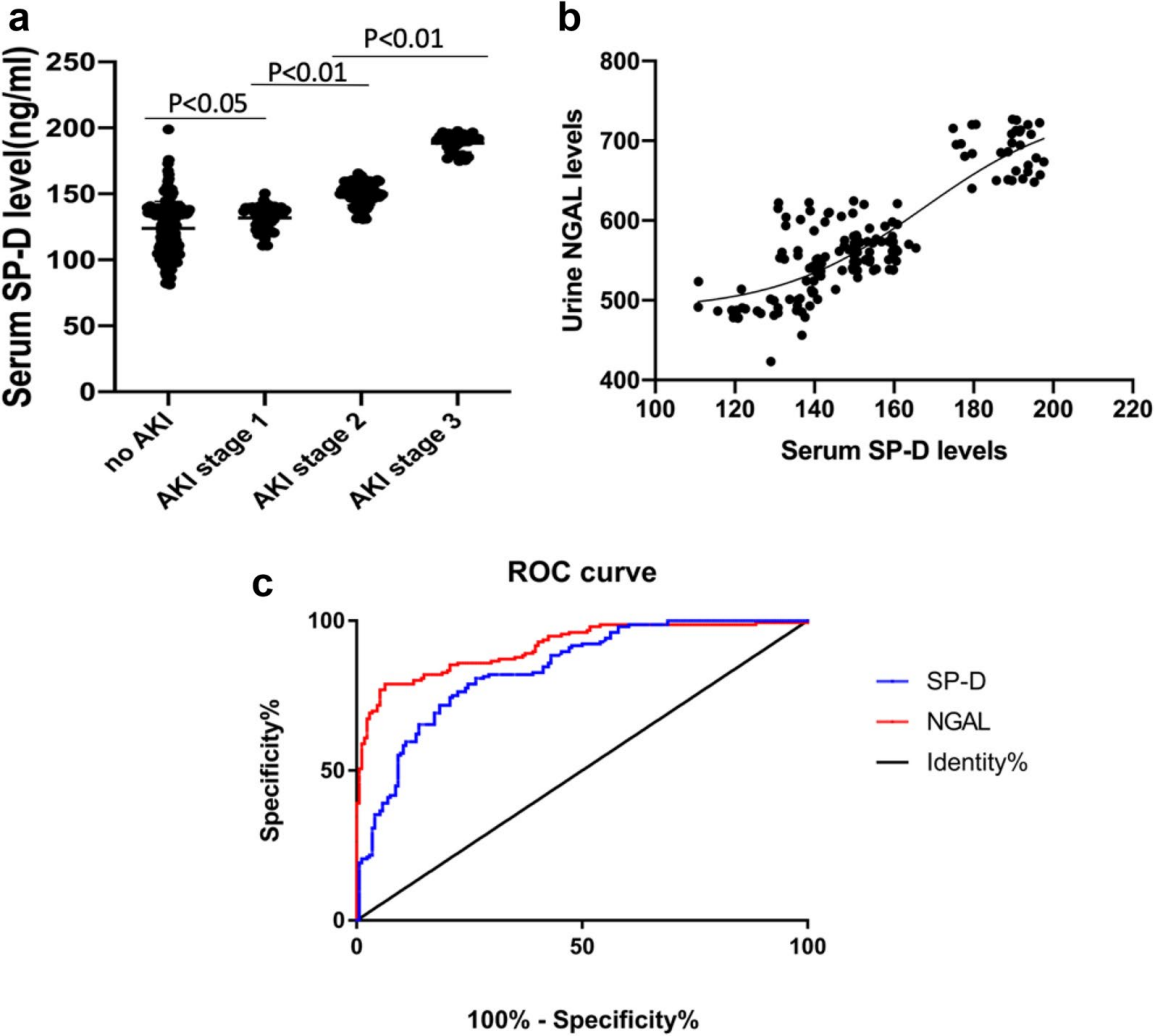
Plasma SP-D and urinary NGAL levels in septic patients. Plasma SP-D levels in septic patients with AKI KDIGO stage 1, 2, 3 and without AKI (**a**). Significantly higher plasma SP-D levels were observed in septic AKI stage 1 patients, which were consistent with the severity of kidney injury. Positive correlation between plasma SP-D levels and urinary NGAL levels were showed (**b**). Pearson analysis shows *r *= 0.85, 95% CI 0.80–0.89. The area under the ROC curve of plasma SP-D level, urinary NGAL level and SP-D-rs721917 CC genotype to identify the presence of AKI were 0.84, 0.91 and 0.58, respectively (**c**)

The average plasma SP-D levels (153 ± 22 ng/mL) and urinary NGAL levels (median: 561, IQR: 536, 611) in septic patients with AKI were significantly higher than in non-AKI septic patients (SP-D levels: 124 ± 20 ng/mL, *P *= 0.001; NGAL levels median: 480, IQR: 469, 496, *P *= 0.001) (Table 1). Furthermore, the average plasma SP-D levels in septic AKI patients significantly increased with severity of KDIGO stage (Fig. 2a). The plasma SP-D levels were correlated with urinary NGAL contents (*r *= 0.85, 95% CI 0.80–0.89, Fig. 2b). The areas under the ROC curve for plasma SP-D levels and urinary NGAL levels to predict AKI were 0.84 and 0.91, respectively. In septic patients who died, plasma SP-D levels (153 ± 23 ng/mL, *P *= 0.001) and urinary NGAL levels (median: 561, IQR: 536, 611, *P *= 0.001) were also increased compared with that in septic patients who survived (Table 4).

**Table 4:** Demographic and baseline characteristics of sepsis patients who died and who survived

Patients	Sepsis		*P*
	Non-survivors	Survivors	
N	N = 78	N = 252	
Mean age, years (SD)	49 ± 10	46 ± 7	0.001^$$^
Male sex, *n*(%)	51 (65)	107 (43)	0.703
Mean SOFA scores (SD)	14 ± 4	10 ± 2	0.001^$$^
Positive blood culture, *n*(%)	23 (30)	46(22)	0.315
Shock, *n*(%)	35 (45)	86(34)	0.387
ARDS, *n*(%)	26 (33)	76(30)	0.583
Comorbidities, *n*(%)			
Hypertension	11 (14)	34(14)	0.533
Diabetes mellitus	15 (19)	38(15)	0.523
Infection foci, *n* (%)			
Respiratory system	36 (46)	106 (42)	0.155
Abdominal cavity	8 (10)	47 (18)	0.640
Urinary tract	9 (12)	62 (25)	0.166
Bloodstream	23 (30)	32 (13)	0.010^$^
Unknown	2 (3)	5 (2)	0.089
Average plasma SP-D, ng/mL	153 ± 23	133 ± 24	0.001^$$^
Median (IQR) urinary NGAL, ng/mL	572 (524, 623)	491 (471, 539)	0.001^$$^

^$^Compared with non-survivors, *P *< 0.05

^$$^Compared with non-survivors, *P *< 0.01

The average plasma SP-D levels of septic patients with AKI with SP-D rs721917CC genotype were significantly higher than those of the other two genotypes (SP-D rs721917CT and TT genotype) (*P *< 0. 05 in both) (Table 5). There was no statistical difference in the median plasma SP-D levels among septic patients with AKI with three rs2243639 genotypes (*P *> 0. 05 in all).

**Table 5:** Plasma SP-D levels of sepsis patients with AKI according to SP-D polymorphisms

Genotypes	Plasma SP-D (ng/mL), Median(IQR)	*P* value
rs721917		
CC	139.3 (124.2, 186.3)	
CT	136.8 (122.6, 149.9)	0.016*
TT	131.0 (106.9, 198.8)	0.001**
rs2243639		
AA	139.4 (113.0, 171.6)	
AG	136.8 (120.7, 150.9)	0.189
GG	135.7 (123.0, 149.9)	0.110

* Compared with CC genotype, *P *< 0.05

** Compared with CC genotype, *P *< 0.01

### Predictors of AKI development and severity and mortality

The multivariate analysis showed that age (OR: 1.139, 95% CI 1.094–1.186; *P* = 0.000), SP-D rs721917CC genotype (OR: 1.996, 95% CI 1.078–3.697; *P* = 0.024), SOFA score (OR: 1.128, 95% CI 1.040–1.225; *P* = 0.004) were independent predictors of AKI (Table 6). There are multiple linear correlations between plasma SP-D levels, urinary NGAL levels and occurrence of AKI. It was not suitable to do multivariate analysis with above two variables.

**Table 6:** Multivariate analysis of predictors of AKI development and severity in sepsis patients

Outcomes	Variables	*P*	OR
Septic AKI vs. no AKI	Age	0.001	1.139
	rs721917CC genotype	0.024	1.996
	Infective foci-urinary tract	0.003	0.358
	SOFA score	0.004	1.128
Septic AKI stage 3 vs. stage 1	Hypertension	0.013	7.979
	Diabetes mellitus	0.010	6.719
Death vs. survival	AKI	0.007	1.260
	Urinary NGAL levels	0.001	1.025
	rs721917CC genotype	0.005	3.695

Furthermore, hypertension (OR: 7.979, 95% CI 1.541–8.675; *P* = 0.013), and diabetes (OR: 6.719, 95% CI 1.581–7.532; *P* = 0.010) were independent predictors of advanced KDIGO stage. The SP-D rs721917CC genotype (OR: 3.806, 95% CI 1.496–7.129; *P* = 0.014) and urinary NGAL levels (OR: 1.025, 95% CI 1.016–1.034; *P* = 0.001) were independent significant predictors of mortality. AKI was also a significant predictor of mortality of septic.

## Discussion

In the present prospective study using data from a large cohort of CCMD septic patients from two tertiary care hospitals in China, we determined *SP*-*D* polymorphisms as well as plasma SP-D levels of these septic patients and examined the association between *SP-D* polymorphisms and the development and severity of AKI in septic patients. We found that the plasma levels of SP-D were significantly higher in AKI patients vs. non-AKI and SP-D rs721917CC genotype was significantly associated with and had a twofold increased risk of AKI. Importantly, we also found that rs721917CC genotype was associated with an approximately fourfold increase in the risk of death in the whole cohort. Our finding suggests that SP-D rs721917CC genotype may be used as a biomarker for AKI development and as a predictor of mortality of septic patients.

Horimasu et al. found that there were racial differences in SP-D polymorphisms [15]. The rate of SP-D polymorphisms rs721917CT genotype and rs2243639AG genotype was 51% and 43%, respectively, in our study cohort, which is similar to a previous report for Chinese population [15]. The SP-D polymorphisms rs721917CC genotype was 30% in our study cohort, which is similar to that (35%) of a healthy Japanese population and higher than that (24%) of a healthy German population in the study by Horimasu et al. We also found that septic patients with AKI had a significantly higher rate of rs721917CC genotype vs. non-AKI patients. This is in agreement with our previous study which found that the frequency of the 11Thr/Thr genotype was significantly higher in patients with AKI than non-AKI patients [9]. The present study also shows that septic patients who died had a higher frequency of SP-D rs721917CC genotype and C allele. Altogether, these findings indicate that SP-D-Thr11Met polymorphism may be a predictor of worse outcome in AKI patients.

We found that septic patients with AKI also had significantly higher plasma SP-D levels vs. non-AKI patients. Further analysis shows that only the median plasma SP-D levels of septic patients with AKI with SP-D rs721917CC genotype were significantly higher than SP-D rs721917CT and TT genotypes while there was no statistical difference among septic patients with AKI with regards to rs2243639A/G genotypes. Our findings are consistent with our previous study [9], while another study found that acute lung injury patients with AKI did not have significantly higher plasma SP-D levels [6], a difference that might be explained by the different underlying diseases in the two populations (sepsis vs. acute lung injury).

Although in the current study SP-D rs721917CC genotype was a significant risk for AKI development, it was not a predictor of AKI severity. Sepsis-induced AKI occurs as a result of undue inflammatory response caused by a large number of inflammatory cells and inflammatory cytokines [16] and a variety of factors, including hemodynamic disorders.

Our study has several limitations. The CKD incidence seemed to be too low in this study. Our general population admitted in this study was regularly young compared with other sepsis populations in the PRISM study. And we could not exclude that patients with unknown CKD who were finally enrolled in our study due to lack of information. The study only examined CCMD septic patients at two tertiary care hospitals in China and did not include patients from the secondary and primary care settings. In addition, though SP-D rs721917CC genotype was a significant predictor of AKI development and mortality of septic patients, the performance of SP-D rs721917CC genotype as a diagnostic or prognostic marker of AKI still needs to be further studied. Moreover, we chose a relatively short time frame for the development of AKI within 30 days of hospitalization. As we were interested in the association between SP-D polymorphisms and AKI and SP-D polymorphisms are stable biomarkers, we believe that the short time frame is reasonable for studying association of SP-D polymorphisms and AKI development and severity. Furthermore, we also found that plasma SP-D levels in sepsis patients with AKI positively correlated with urinary NGAL levels, suggesting that combined use of SP-D polymorphisms and plasma biomarkers would increase diagnostic and prognostic efficacy for sepsis-induced AKI.

In conclusion, our study demonstrated that SP-D rs721917CC genotype is an independent and significant predictor of AKI development and mortality of septic patients. SP-D rs721917C/T polymorphisms should be further studied as a diagnostic and prognostic biomarker to facilitate early recognition of AKI so that treatment can be instituted promptly.

## Conclusion

Our study showed that increased plasma SP-D level is associated with a higher risk of AKI in patients with sepsis. The SP-D rs721917CC genotype is an independent and significant predictor of AKI development and mortality of septic patients. The SP-D rs721917C/T polymorphisms should be further studied as diagnostic and prognostic biomarkers to facilitate early recognition of AKI.

## Data Availability

The datasets supporting the conclusions of this article are included within the article and its additional file.
